# Supplementary figures and images for: BigBrain 3D atlas of cortical layers: Cortical and laminar thickness gradients diverge in sensory and motor cortices
Source: PLoS Biol. 2020 Apr 3;18(4):e3000678. doi: 10.1371/journal.pbio.3000678 (PMC7159250; doi:10.1371/journal.pbio.3000678)

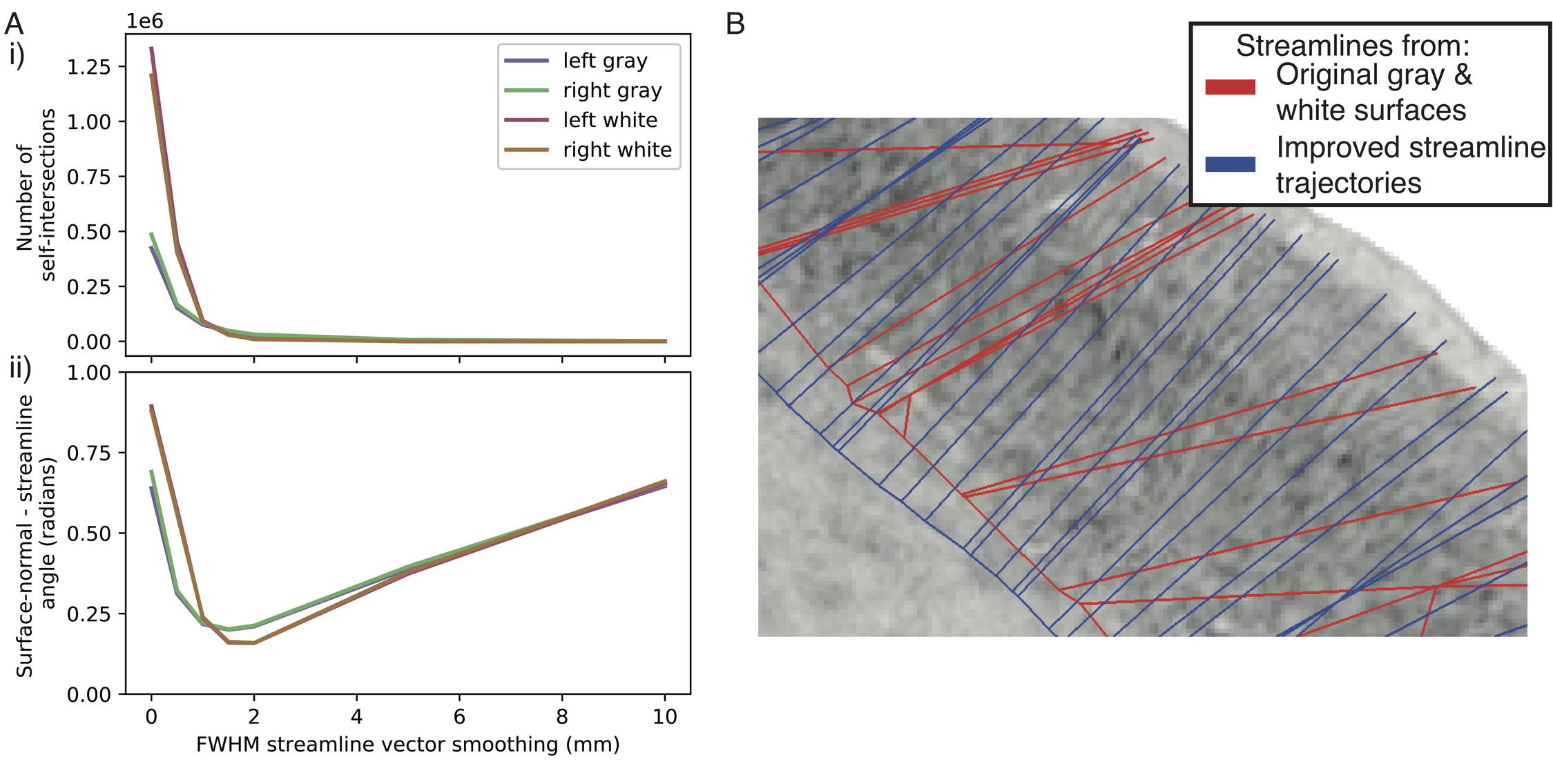

Supplement: S1 Fig — (A) Streamline vectors were smoothed across the cortical surface by varying degrees to assess the impact of smoothing on (1) the number of self-intersections in the pial and white surfaces and (2) the angle between the streamline and the normal vector on the pial and white surfaces. These optimization curves demonstrate that a FWHM of around 2 mm drastically decreases the number of self-intersections and obliqueness of the streamline vectors relative to the pial and white surfaces. (B) Visualizing streamlines against a histological section. Streamlines more closely follow visible cortical columnar trajectories after this improvement (blue) relative to before this streamline vector smoothing process (red). FWHM, full width at half maximum. (TIF) [file pbio.3000678.s001.tif]

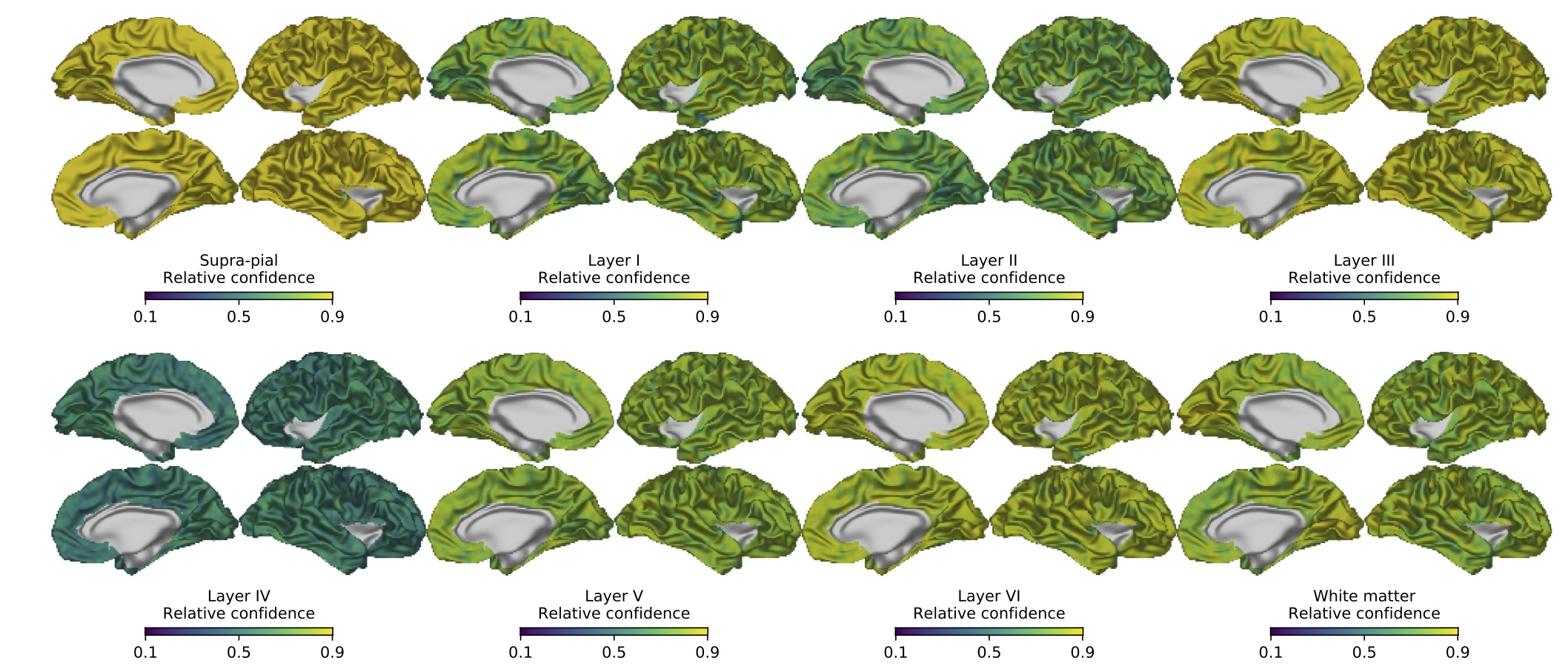

Supplement: S2 Fig — Per-vertex confidence is defined as the difference between the prediction value for the highest predicted class and the value of the second-highest predicted class, averaged over the whole profile. This gives an approximation of the reliability of laminar segmentations for the cortex where ground truth manual segmentations have not been carried out. Confidence for suprapial and white matter classes was high throughout the cortex, thus increasing the confidence in overall cortical thickness measures. Layers exhibit relatively consistent confidence maps, with layer IV least confident overall. This pattern matches with visual observations that layer IV is the most difficult to identify. Regional variations in confidence can guide the choice of target regions for future extensions to the training data. (TIF) [file pbio.3000678.s002.tif]

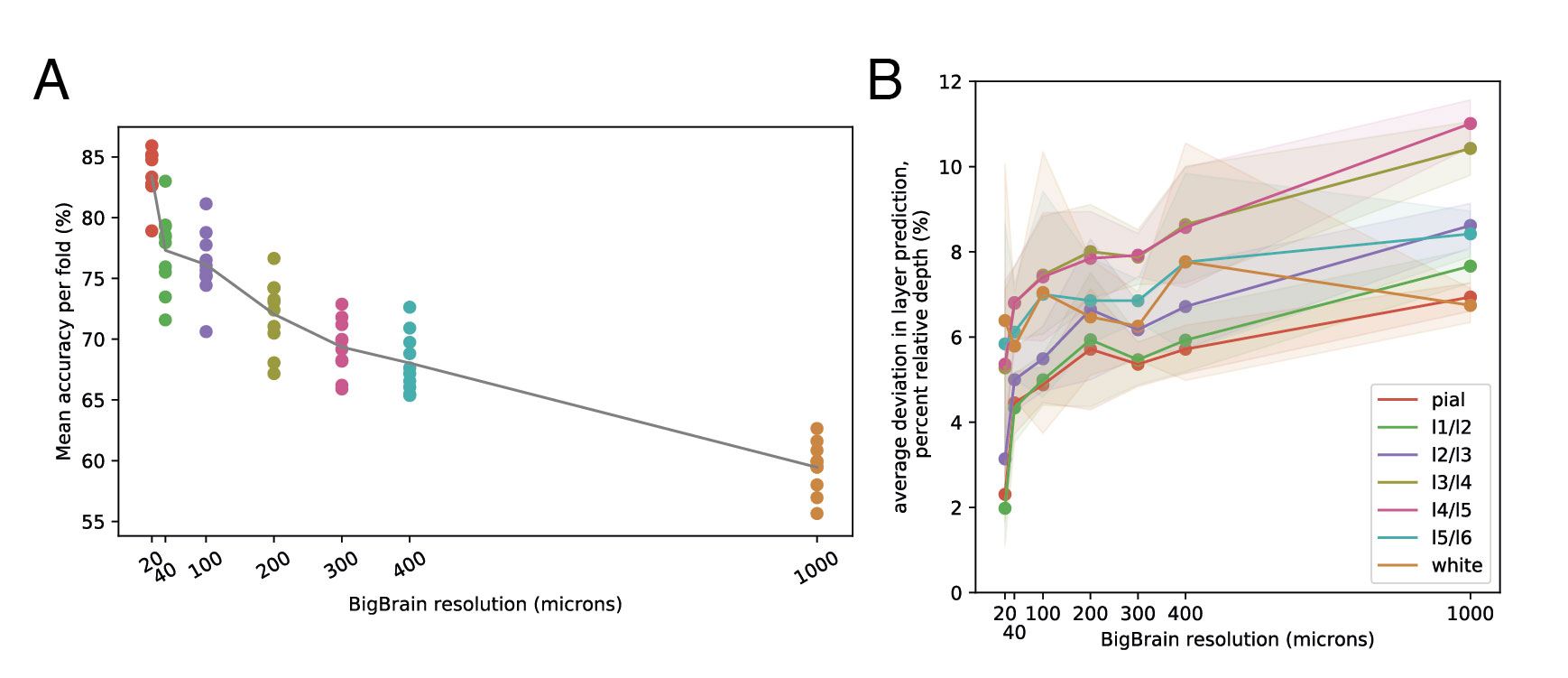

Supplement: S3 Fig — (A) Overall per-point accuracies on withheld test regions calculated using 10-fold validation. Accuracy decreases with decreasing resolution. (B) Mean deviation in depth prediction on test folds between prediction and manually defined layers. Pial/layer I and layer I–II boundaries exhibited the smallest deviations, followed by II/III, with layer III/IV and VI/white boundaries exhibiting larger deviations. (TIF) [file pbio.3000678.s003.tif]

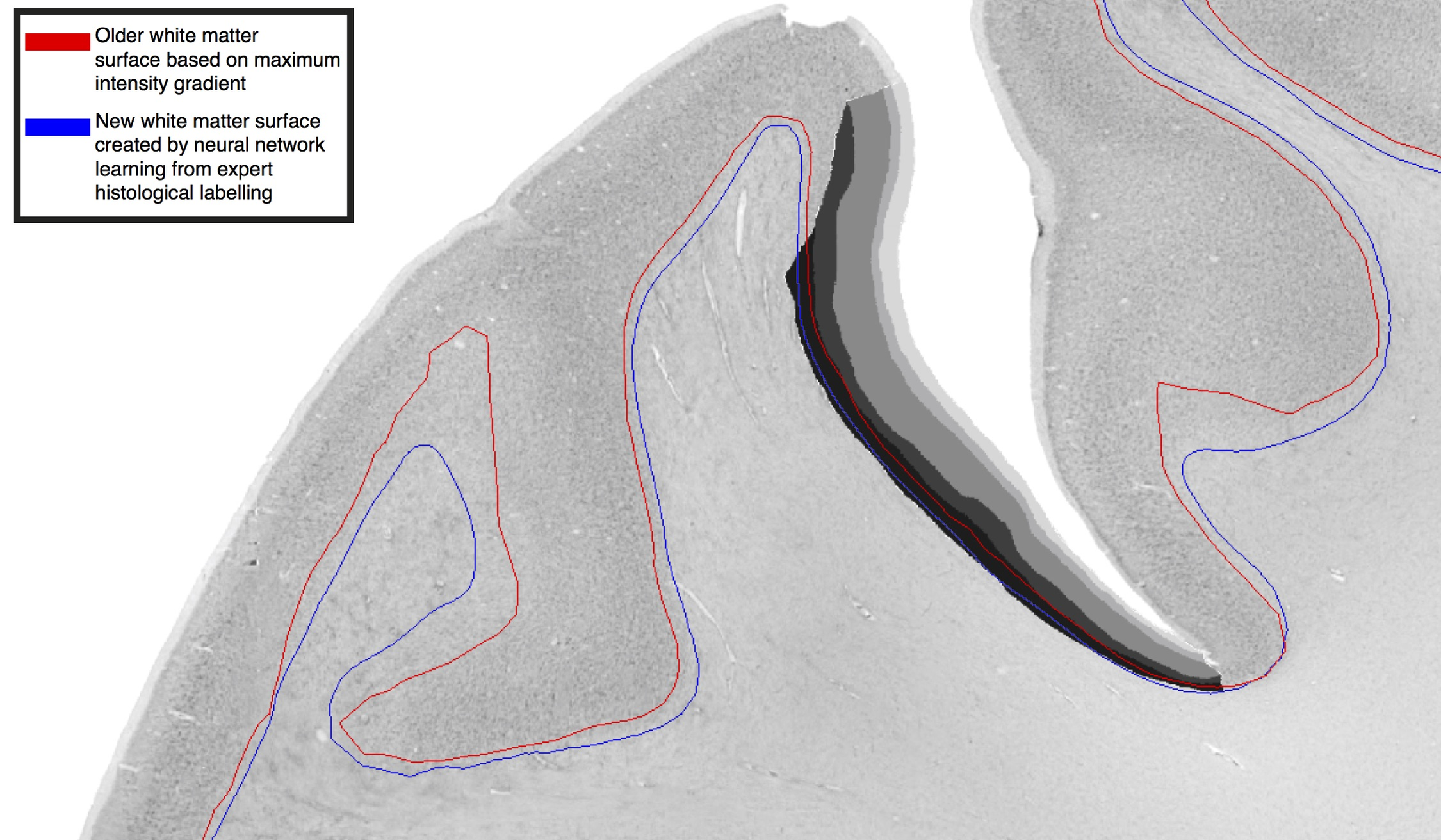

Supplement: S4 Fig — For visual comparison, the surfaces are overlaid on a 2D section, in which manually segmented layers are available. The maximum intensity gradient white surface (red) was identified on lower-resolution data (200 μm). Superimposed on the histology in grayscale is a section of cortex in which 6 layers were manually segmented. The automated blue surface follows the manually delineated gray–white matter boundary, which is determined by the presence of cortical neurons. By contrast, the red (maximum gradient) surface follows a feature that is consistently superficial to the gray–white matter boundary, corresponding to the layer VIa/VIb boundary. This systematic difference highlights the role of using histological expertise when translating across scales and fields to ensure consistent definitions. It also raises an important question on the placement of the white surface in MRI cortical reconstructions, which is placed at the maximum MRI intensity gradient. This gradient is determined predominantly by myelin contrast and therefore influenced by changes in interregional and longitudinal in cortical myelination. Future cortical segmentation algorithms need to be developed with close reference to histological definitions of the gray/white boundary. (TIF) [file pbio.3000678.s004.tif]

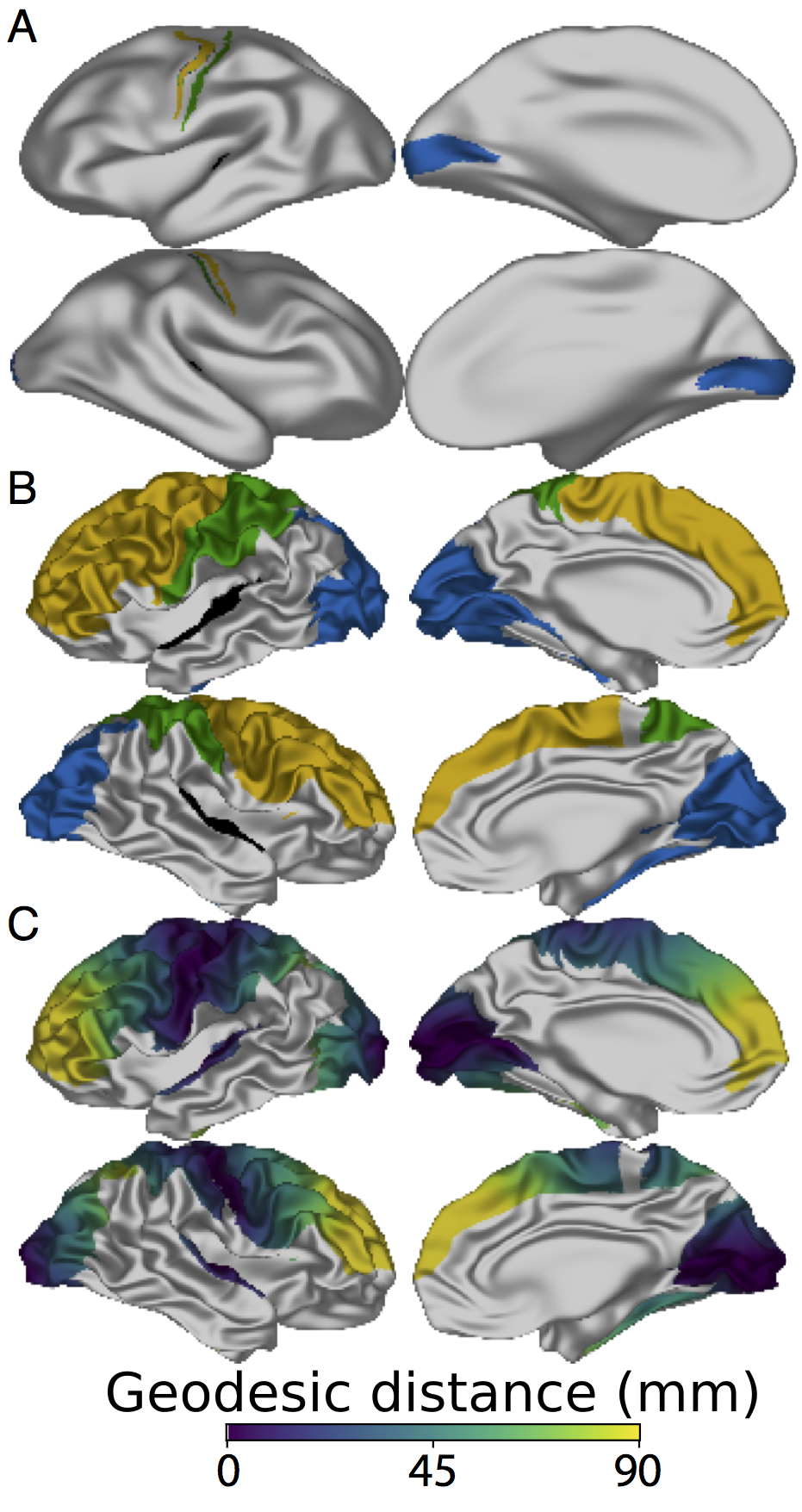

Supplement: S5 Fig — (A) Manually segmented primary visual (blue), primary auditory (black, partially buried in the lateral sulcus), primary somatosensory (green), and primary motor (yellow) areas, projected onto a heavily smoothed surface. (B) Manually segmented regions across which cortical and laminar hierarchical thickness gradients were calculated. (C) Geodesic distance across the cortical surface from the primary areas. (TIF) [file pbio.3000678.s005.tif]
