# Supplementary material for: BigBrain 3D atlas of cortical layers: Cortical and laminar thickness gradients diverge in sensory and motor cortices
Source: PLoS Biol. 2020 Apr 3;18(4):e3000678. doi: 10.1371/journal.pbio.3000678 (PMC7159250; doi:10.1371/journal.pbio.3000678)
Supplement: Attachment — Submitted filename: BigBrain_PLOS_response.docx [file pbio.3000678.s008.docx]

Response to reviewers.

Reviewer #1: This is an interesting manuscript from a well established, expert group with solid credentials in the domains of cortical organization, cytoarchitectonics, and atlas presentation. The manuscript continues and extends related investigations (as, K. Wagstyl et al., Cerebral Cortex, 2018), but can be considered as a significant contribution to this still developing field, and providing specific technical improvements.

We thank the reviewer for this assessment of the paper and the group’s broader work.

The latter, as pertain to a comparison of manual vs. automatic segmentation of laminar borders and thickness gradients , and the calculation of laminar thickness over the whole brain. I have only a few comments about issues I think would benefit from author consideration and revision.

1. Primarily, the figures. At first glance, these are attractive, but with closer inspection, I found the figures to be uneven in quality. In Figure 1, the very important issue of laminar borders is not successfully conveyed, largely owing to too low a magnification. The blue of layer II was hard to distinguish from layer I, and the tan of Layer V, with only a few exceptions, was indistinguishable from layer VI. Can the authors 1) modify the color code? and/or 2) re-format the figure. The section outlines could easily be smaller, with more space given to higher magnification insets, where the laminar differentiation (as in Figure 2) can be more fully appreciated.

We thank the reviewer for these suggestions. We have changed the colour code to improve interlaminar contrast and reformatted the figure to show the sections at higher magnifications. This has helped to visualise individual layers on the sections.

In Figures 3 and 4, the coloration is hard to appreciate (even though closely following that in Wagstyl et al., Cerebral Cortex, 2018). Can the authors consider modifying the contrast or other aspect?

Following the reviewer’s suggestion, the color scale has been changed for both figures 3 and 4 and the thresholding in figure 3 has been modified to improve colour contrast.

2. There is a needless lack of clarity regarding "sensory cortices." For most of the manuscript, attention is given to visual and somatosensory, but the authors obviously included auditory cortex in their sensory cortical group (Conclusion, and page 14).

The reviewer is absolutely correct that sensory cortices was ambiguous and auditory cortex occasionally missed from the list of included systems. We have amended the introduction to clarify this in two places:

*MRI approaches have demonstrated patterns of cortical thickness relating to functional and structural hierarchical organisation across visual, somatosensory and auditory cortices of both macaques and humans* [*[10]*](https://paperpile.com/c/nvEgXa/UWnl6)*.*

And

*We therefore set out to automate segmentation of cortical layers in 3D in order to characterise patterns of cortical and laminar thickness across visual, somatosensory, auditory and fronto-motor cortical areas.*

As well as rectifying the omission in the results and conclusions:

*Consistent with previous findings using in vivo MRI* [*[10]*](https://paperpile.com/c/nvEgXa/UWnl6) *and 2D histological measurements* [*[8]*](https://paperpile.com/c/nvEgXa/Uw9R)*, visual, somatosensory and auditory hierarchies exhibited a gradient of increasing cortical thickness from primary sensory to higher order areas. These gradients were primarily driven by layers III, V, VI.*

&

*In the visual, somatosensory and auditory cortices, an increasing gradient of histological cortical thickness was identified and found to be primarily driven by layers III, V and VI.*

3. There is some lack of detail; for example, relating to sulcal and gyral components. Area V1 included multiple loci for both. Can this be elaborated on?

We understand the reviewer to be referring to the area of text describing the choices of manually annotated training regions.

We have extended the description in the methods section as follows, including a link to download all of the annotated data.

*These regions were chosen to give a distribution of examples demonstrating a variety of cytoarchitectures, a variety of rostro-caudal locations, in both gyri and sulci, from sections where the cortex was sectioned tangentially.*

*Layers were segmented according to the following criteria. Layer I, the molecular layer, is relatively cell-sparse with few neurons and glia. Layer II, the external granular layer, is a much more dense band of small granular cells. Layer III, the external pyramidal layer, is characterised by large pyramidal neurons that become more densely packed towards its lower extent. Layer IV, the internal granular layer (usually referred to simply as the “granular layer”), generally contains only granular neurons, bounded at its lower extent by pyramidal neurons of layer V. Layer V, the internal pyramidal layer, contains large but relatively sparse pyramidal neurons while layer VI, the multiform layer, has a lower density of pyramidal neurons* [*[1]*](https://paperpile.com/c/nvEgXa/aQCV)*. Alongside association areas with such typical neocortical laminar structure, samples from the primary visual and motor cortices were specifically included as they exhibit unique laminar characteristics.*

*...*

*This 1D profile-based approach greatly expanded the training dataset from 51 labelled 2D samples to over 500,000 profiles. Coregistered manually annotated data is available to download at ftp://bigbrain.loris.ca/BigBrainRelease.2015/Layer_Segmentation/Manual_Annotations/.*

4. There is some "over-reaching." This occurs repeatedly in the Discussion: Paragraph 2 "the cortical microcircuit": the Authors have inadequate data (interneurons; EM) for commenting on cortical microcircuitry. Paragraph 2 (page 7) "Likely driver of the laminar and overall thickness gradients" = ? Same, page 8: "cortical microcircuit properties" = ? I suggest that the comparisons with lamination in MRI and Big Brain are all substantive and important to state, but some of these other points, in my opinion, are not compelling and thus distracting.

We agree that the confidence of the discussion of cortical microstructure and especially microcircuit properties were overstated given the data actually analysed. However we do think that hypotheses relating the underlying histology to cortical and laminar thickness gradients do warrant some consideration. We have therefore rephrased the discussion paragraphs into more tentative language and removed all references to ‘microcircuits’.

*Gradients of thickness are large scale markers of systematic changes in the cortical histology. The volume of the cortex is 80-90% neuropil* [*[33–37]*](https://paperpile.com/c/nvEgXa/zoSj+bhFC+bCIEe+Mv4M+CUPOb)*, of which 60% is axons and dendrites and the remainder is synaptic boutons, spines and glia. As neuronal density decreases with increasing cortical thickness* [*[38,39]*](https://paperpile.com/c/nvEgXa/lGr3Y+aEG7y)*, and most of the volume of the cortex is neuropil, increased thickness is most likely to mark increased intracortical connectivity* [*[7]*](https://paperpile.com/c/nvEgXa/8qeJE)*. At a laminar level, the strongest contributors to the overall thickness gradients were layers III, V and VI (Figure 6). Cell-morphological studies in macaques have shown that the cell size and dendritic arborisation of layer III and V pyramidal neurons increase along the visual pathway* [*[18,40,41]*](https://paperpile.com/c/nvEgXa/JNwlb+dQTeW+3aJVB)*. Similarly, afferent axonal patch sizes scale with pyramidal neuronal arborisation* [*[42]*](https://paperpile.com/c/nvEgXa/hj5Qm)*. Increases in dendritic arborisation, axonal field size and number of synapses would all give rise to an increase in the volume of laminar neuropil, and are therefore a plausible contributors in the laminar and overall thickness gradients measured here. Gradients of layer thickness provide us with a meso-scale link between in vivo patterns of MRI cortical thickness and histological changes in the cortical structure. Such links help us to understand the neurobiological significance of interindividual, longitudinal and neuropathological biomarkers*[*[7]*](https://paperpile.com/c/nvEgXa/8qeJE)*.*

And

*Thus there is a coherent group of cortical histological properties which diverges from patterns of cortical myelination and fMRI-derived gradients, establishing the motor cortex at the peak of a gradient of increasing cortical thickness, layer III, V and VI thickness and pyramidal neuronal arborisation, with primary sensory areas at the opposite extreme.*

Reviewer #2, Matthew F Glasser: The authors conduct a very interesting analysis of the cortical layers of the big brain dataset, using machine learning to automate the problem and find results that are in agreement with prior datasets and my neuroanatomical expectations. I have a few minor comments that I hope the authors are willing to address.

We thank the reviewer for this complementary summary of our paper.

1)	Was the mid-surface constructed according to the equal volume principle (Bok et al 1929, Van Essen et al 1980 Journal of Comparative Neurology, Waehnert et al 2014 Neuroimage)?

The mid-surface in question was only used to initialise vertex positions prior extraction of streamlines. It was not constructed according to the equal volume principle, but instead almost exactly the opposite. In the equivolume surface the mid-surface is closer to the surface with *lower curvature*. This causes a problem when trying to find corresponding locations in a sulcus on the pial surface without streamlines intersecting. Instead this non-intersecting mid surfaces was created to be close to the pial surface in sulcal fundi and close the white surface in gyri. This mid-surface was only used for initialising trajectories of vertex streamlines and not used in any subsequent analysis. This point has been clarified in the methods:

*For initialising vertex locations, a mid-surface was generated that was closer to the pial surface in sulci and closer to the white surface in gyri, weighting the distance vector by the cortical curvature. Thus, to prevent intersections of subsequent vertex vectors, the mid-surface was closer to the surface with the higher curvature.*

2)	To correct for shrinkage, it is said that the big brain was registered to MNI space. Glasser et al 2016 Nature Neuroscience pointed out that MNI space is "drifted" relative to the average of the individual brains prior to registration, leading to an overestimation of 37% brain volume (see supplementary Figure 9). Was this corrected for or avoided somehow or avoid biasing the results presented here?

We thank the reviewer for this observation, which we had previously not addressed. On closer reading of Glasser et al 2016, the overestimation is introduced by non-rigid transformations to the aligned space. Our registration was limited to the linear scaling, which closely corresponded to the independent estimations of shrinkage using histological brain-weight techniques. We have amended the text to acknowledge this consideration in the registration process.

*This gave a volume-based (3D) shrinkage factor of 1.931, which corresponds to an isotropic length-based (1D) shrinkage factor of 1.245.*

*To estimate the scale of shrinkage in each of the three orthogonal directions, the BigBrain volume was linearly coregistered to a volumetric MRI template derived from a group of older subjects (ADNI)* [*[58]*](https://paperpile.com/c/nvEgXa/Rx2w)*. The transformation matrix gave linear scale factors of 1.15, 1.22 and 1.43 in the x,y and z directions, with a mean of 1.26. The concordance of these measures of shrinkage suggests that subsequent thickness and length estimates can be adequately corrected for comparison to in vivo measures.*

*Thus, to approximately compensate for the non-uniform compression of xyz, we transformed the mesh surfaces into MNI space based on the ADNI template. Subsequent thickness analyses were carried out on the transformed meshes. Such compensation for shrinkage is necessary when analysing cortical thickness gradients on oblique profiles in 3D over the whole brain. Non-linear corregistration was not applied as this can lead to localised warping and non-biological thickness measurements*[*[59]*](https://paperpile.com/c/nvEgXa/8ZVU)*.*

3)	The figures showing overall and laminar cortical thickness might be better displayed using more inflated surfaces so that one can see further inside the sulci. Additionally, the color scales are not very dynamic, making it harder to appreciate the differences across the cortical surface.

Following the reviewer’s suggestion, the color scale and thresholding have been modified to improve colour contrast in figures 3 and 4. Additionally the surfaces were inflated further to allow better visualisation inside the sulci. We have still retained some of the gyral-sulcal structure in place as we find such markers useful for visual orientation.

4)	The authors point out a potential mismatch between MRI-based white matter surfaces and the true anatomical white matter surface based on the mismatch between the big brain's original white matter surface and the improved one using machine learning. While it is true that a maximum intensity gradient is typically used to place the grey/white boundary, the source of this gradient would be different in T1w or T2w MRI versus the cell-body stained histological data here. Thus, it is not a given that MRI surfaces will have the same bias as shown here. Instead, I would recommend the authors explicitly compare group average cortical thickness maps made from high quality T1w and T2w data from the HCP to their gold standard thickness maps to identify any global or local biases.

In response to the reviewer’s suggestion, we have now included a comparison with the HCP average cortical thickness map taken from 1200 individuals. Overall there was a good agreement (r~0.7) such that MRI thickness variability mostly reflects histological variability. Areas that appeared thinner than expected in MRI were heavily myelinated primary areas (eg 3b and V1). Interestingly, the insula (AAIC PoI1 PoI2) were all overestimated in MRI. This may be as insular cortex is thin but highly folded thus accurately following the convolutions with MRI resolution is a challenge.

Figure 3 has been extended to include this comparison and the methods and results have been amended as follows:

*Methods*

*For comparison, several existing surface-based parcellations of human cortical surfaces (von Economo:* [*[8,62]*](https://paperpile.com/c/nvEgXa/Uw9R+lsGT)*; Glasser:* [*[63]*](https://paperpile.com/c/nvEgXa/ezMbV)*) were coregistered to the BigBrain cortical surfaces using an adaptation of the Multimodal Surface Matching approach* [*[64,65]*](https://paperpile.com/c/nvEgXa/O4cG+yQk1)*. These parcellations enabled comparison with histological data from von Economo and in vivo cortical thickness measurements from the Human Connectome Project.*

*Results:*

*BigBrain histological thickness values also closely correlated with regional values of MRI cortical thickness (Figure 3C). Overall regional values were highly correlated (left hemisphere: r=0.62, right hemisphere: r=0.75). Differences may relate to individual variability, age differences and modality-specific biases. In a ranking of the differences between measurements, MRI cortical thickness was thinner than expected for heavily myelinated primary sensory areas (LBelt, 3b and V1), but thicker than expected for insular and peri-insular regions. This may be as the cortex is thin but heavily convoluted, with sulci that are fused difficult to resolve using MRI. Thickness used in the histological and MRI comparisons can be found in Supplementary tables 1 & 2.*

*Figure 3 caption:*

*Comparison of cortical thickness from the BigBrain with von Economo and Koskinas histological measurement and MRI cortical thickness data from the Human Connectome Project* [*(Glasser et al. 2016)*](https://paperpile.com/c/nvEgXa/ezMbV)*. Thickness values range from 1.8mm in the calcarine sulcus to 4.5 mm in the precentral gyrus. A) Per-vertex cortical thickness values from the BigBrain (displayed on smoothed surfaces, values were smoothed 3mm FWHM). Thicker regions of cortex included the precentral gyrus containing the primary motor cortex. By, the occipital cortex around the calcarine sulcus was particularly thin. Also visible are smaller scale variations in thickness than can only be observed through such high-density measurement. Von Economo reported thickness measurements from around 50 cortical areas, whereas the thickness of around 1 million vertices has been measured on BigBrain. B) Regional BigBrain thickness values were highly correlated with measurements from von Economo and Koskinas. The size of each point is proportional to the area of the cortical region and overall correlations were weighted according to these areas. The precentral gyrus, area FA was the area of greatest discrepancy where BigBrain provided a lower estimate than von Economo. This might in part have been due to averaging of many vertices across the precentral gyrus in BigBrain, in comparison to a single measurement made by von Economo. C) Regional BigBrain thickness values were also highly correlated with MRI cortical thickness values. MRI thickness appears to be overestimated in the insula, where it is thin in both histological datasets. This may be as the insula is highly convoluted and thus challenging to accurately follow at lower resolutions.*

Reviewer #3: The authors present a novel application of machine learning to neuroanatomy of the human cerebral cortex. The algorithm used could segment cortical layers based on training data from drawings from human experts. The results focus on assessments of laminar and overall cortical thickness to construct a 3D laminar atlas of the human cerebral cortex. Both layer-specific and overall thickness estimates correlated well with the estimates of von Economo, with only layer 2 measurements showing weak agreement. The authors concluded that somatosensory and visual cortical systems showed opposite thickness gradients to the fronto-motor system.

The constructed atlas of the human cortex at laminar resolution will be useful for others who may use the algorithm to map other findings based on markers to shed light on the organization of the human cortex.

We thank the reviewer for this description of the manuscript.

Several points throughout the manuscript need clarification. Despite the methodological nature of the paper, the Methods are sketchy and need to be elaborated.

It is not clear what stains the authors used to measure the layers from histological sections.

We acknowledge the information in the methods section is currently incomplete. Much of the detail is found in the original presentation of the BigBrain dataset (Amunts et al., 2013, *Science*) and is beyond the scope of this manuscript. Nevertheless, we will endeavor to clarify where necessary. The stain was a Merker silver stain for cell bodies. We have adapted the methods to include this information:

*BigBrain is a 20x20x20μm (henceforth described as 20μm) resolution volumetric reconstruction of a histologically processed post mortem human brain (male, aged 65), where sections were stained for cell bodies*[*(Merker 1983)*](https://paperpile.com/c/nvEgXa/0Sn5)*, imaged and digitally reconstructed into 3D volume*[*(Amunts et al. 2013)*](https://paperpile.com/c/nvEgXa/vid5)*.*

In addition, in view of the exclusion of some areas from the measurements, either by choice or because of methodological difficulties, the caveats should be discussed, and conclusions tempered.

Major comments:

1. p 13, the authors stated: "Manual masks were created to remove the medial wall, the allocortex, including parts of the cingulate and entorhinal cortex which do not have 6 layers, and large cuts in the anterior temporal cortex (caused by the saw during extraction of the brain from the skull) from subsequent analyses."

This is an important point and should be clearly stated in Results as well.

We thank the reviewer for highlighting this lack of clarity over masking and have added the following text to the results and methods sections.

*Results*

*Vertices located in the medial wall and temporal lobe cuts were masked for all analyses of cortical thickness, while allocortex was additionally excluded for analyses of laminar thickness.*

*Methods*

*Manual masks were created to remove the medial wall and small numbers of vertices located in the large cuts in the anterior temporal cortex (caused by the saw during extraction of the brain from the skull) from subsequent analyses. The allocortex, including parts of the cingulate and entorhinal cortex which do not have 6 layers, were excluded for comparisons of laminar thickness with von Economo measurements.*

It is often difficult to distinguish layer 2 from layer 3, and layer 5 from layer 6 in several association areas. The Results need to be expanded to include commentary on this issue.

3. Page 6, under "Confidence results", "layer IV is often the most difficult to identify", is also related to the point above, because many areas either lack or have a poorly delineated layer IV. The authors unfortunately did not include many of these areas in the analysis, but even so, layer IV varies considerably among association areas that have six layers.

We agree that these borders can be particularly difficult to reliably distinguish. We have added the following paragraph to the results to discuss issues in identifying layer IV and layer II and III. This also includes a reference to the supplementary figure in question.

*The reason for the lack of agreement between layer II measurements might arise from known difficulties in distinguishing a clear boundary between layer II and layer III alongside a low amount of interareal variation in layer II thickness. Similar challenges exist in identifying layer IV and the layer V VI boundaries exist in many cortical areas, which may account for some of the discrepancies between the two sets of laminar measurements. These difficulties might be reflected in the lower ‘confidence’ values associated with these areas (Supplementary Figure 2).*

*Nevertheless, despite the challenges associated with manual laminar segmentation and the fact that measurements were made on different individuals nearly a century apart, there are high overall correlations between these two laminar atlases.*

2. Related to the above, there is an incorrect statement in the first sentence of the Introduction "The cerebral cortex has six cytoarchitectonic layers..". As classical and modern studies have clearly shown, many cortical areas have fewer than six layers; these include the cortical limbic areas, as shown in the classical studies of von Economo/Koskinas and Sanides, which the authors refer to. More recent studies have relied on the systematic variation of the cortex to derive principles of cortical organization and connections.

The reviewer is correct in pointing this out. We have changed the first sentence to read as follows:

*The cerebral cortex has laminar cytoarchitectonic structure that varies depending on cortical area*[*[1]*](https://paperpile.com/c/nvEgXa/aQCV)*, and cannot readily be resolved using in vivo MRI techniques*[*[2]*](https://paperpile.com/c/nvEgXa/4V9k)*.*

4. In the Abstract: "In contrast, fronto-motor cortices showed the opposite pattern, …", please re-work the sentence to state the direction of "decreases in total and pyramidal layer thickness".

Thank you for highlighting this lack of clarity. The text now reads:

*In contrast, motor-frontal cortices showed the opposite pattern, with decreases in total and pyramidal layer thickness from motor to frontal association cortices.*

5. In the Abstract, the authors should state that the BigBrain data are derived from a single postmortem human brain. In view of this fact, the reference to '..a 3D model of the human brain..' should more appropriately be called a 3D atlas.

We agree with the authors that ‘model’ does not fully clarify that it is a single brain. We have amended the text as follows:

*It was derived from a 3D histological atlas of the human brain at 20 micron isotropic resolution (BigBrain), using a convolutional neural network to segment, automatically, the cortical layers in both hemispheres.*

6. Defining "geodesic distance" at the first instance of use is necessary for a general audience of readers who may not be familiar with graph theory.

This is indeed a technical term that was not clearly defined. The text now reads:

*Topographically, hierarchies are organised such that progressively higher cortical areas are located with increasing geodesic distance (the shortest path traversing the cortical surface) from their primary areas* [*[10,27]*](https://paperpile.com/c/nvEgXa/UWnl6+RVFDR)*.*

7. One of the limitations of the study that deserves discussion is the sole reliance on laminar and overall cortical thickness. Many architectonic areas have gyral and sulcal parts, and the former are generally thicker than the latter but are not necessarily less dense. The authors have not used density as an additional measure but refer to studies from the literature for discussion of this point. However, the statement that "..neuronal density decreases with increasing cortical thickness [38,39]", (page 7), is not based on detailed histological data of many areas. The use of a limited number of areas, lumping together areas that differ in thickness, or using low-resolution data, may not critically test the generality of a trend or model. This is evident in data on laminar-specific connections from the literature that were advanced as supportive of hierarchical or other models, but held only for a limited set of areas, or held only when some data were excluded from analysis, as was the case in ref. 20.

We absolutely agree that the use of limited number of areas and lumping together areas may not test the generality of a trend. This is in fact a major motivating factor in creating this atlas. Rather than using 30-50 coarse areas, the thickness and laminar values are densely sampled with 500,000 samples per hemisphere.

Unfortunately there is a limitation that density measurements have been referred to elsewhere in the literature, rather than measured directly. The reason for this is that the full 3D bigbrain is 20micron resolution, insufficient for delineating cell bodies. Unfortunately, staining intensity and cell density are confounded by variations in cell body size that preclude the simple use of intensity for quantitative density estimates.

We have added a discussion of this to the limitations:

*Finally, at 20 micron resolution, individual neuronal cell bodies cannot readily be resolved. Future work to generate comprehensive histological atlases with even higher resolution will offer further insights into the direct links between meso-scale features presented here and micro-scale histological properties.*

Nevertheless, the main argument in the paragraph neuronal density is that changes in other cortical properties, rather than increases in neuronal density, are the primary driver of cortical thickness increases. We have therefore reworded the sentence to reflect more caution surrounding the density & thickness relationship:

*As neuronal density does not tend to correspond with increases in cortical thickness* [*[38,39]*](https://paperpile.com/c/nvEgXa/lGr3Y+aEG7y)*, and the majority of the cortical volume is made up of neuropil, increased thickness is most likely to indicate increased intracortical connectivity* [*[7]*](https://paperpile.com/c/nvEgXa/8qeJE)*.*

Figures:

*8. Fig. 1. It is not possible to distinguish layer layer I from II or III with the colors chosen; a brighter color between layers would help.*

We have changed the colour scheme and enlarged the images in Figure 1 to help readers visualise and distinguish the layers in detail.

9. Fig. 3. It would be helpful to identify areas that show the greatest disagreement with the von Economo data.

We thank the reviewer for this suggestion. Figure 3 has now been extended to include a comparison with both von Economo and MRI HCP data. In the figure caption, we have now described the areas of greatest disagreement for both datasets and included supplementary tables with all of the extracted values for greater transparency.

Figure 3 caption

*B) Regional BigBrain thickness values were highly correlated with measurements from von Economo and Koskinas. The size of each point is proportional to the area of the cortical region and overall correlations were weighted according to these areas. The precentral gyrus, area FA was the area of greatest discrepancy where BigBrain provided a lower estimate than von Economo. This might in part have been due to averaging of many vertices across the precentral gyrus in BigBrain, in comparison to a single measurement made by von Economo. C) Regional BigBrain thickness values were also highly correlated with MRI cortical thickness values. MRI thickness appears to be overestimated in the insula, where it is thin in both histological datasets. This may be as the insula is highly convoluted and thus challenging to accurately delineate at lower resolutions.*

10. Figure 4: A table with the quantitative laminar thickness data is needed in Results. A correlation matrix displaying the agreement between the current results and the von Economo findings would be easier to parse than the brain surface heatmaps. Something similar may help with Supplementary Figure 2.

We thank the reviewer for this suggestion. We agree that a table of thickness values is an essential addition and have therefore included two additional supplementary files of parcellated thickness data. These are parcellated according to the von Economo and Human Connectome Project MMP parcellation (Glasser et al., 2016) (Supplementary data 1 & 2).

In addition, on Figure 4, correlation values across all layers have been included above the color bars to quantify the agreement across the layers.

11. Fig. 6A is confusing; does the label under 'motor' include other areas, and if so, which?

References to "Fronto-motor" (and throughout the manuscript) conjure up the opposite image of the one intended; perhaps it would be better to say motor-frontal.

We thank the reviewer for this observation. Motor-frontal better captures the pattern we are describing so we have changed all references from “fronto-motor” to “motor-frontal”, throughout the text. Likewise for figure 6A, the reference has been changed to “motor-frontal”. Regions included in each of these systems are reported in Supplementary figure 5B.

Is layer III in motor cortex thicker than layer V, as in D and E?

We have double checked this in response to the reviewers questions. From BigBrain and von Economo laminar thickness data (Figure 4 layer III, Figure 6A & B), layer III is thicker in the motor cortex than layer V (FA III=1.26mm, V=0.85mm). The schematic diagram for 6E may not reflect this proportional changes precisely to scale, but the general patterns reflect our findings.

12. As also mentioned above, the inverse relationship in the motor cortex that the authors describe is partly driven by the areas included in the study. A detailed list of all areas that were included in the analysis is needed.

We agree that clarity is required over which areas we included in analyses of motor and sensory areas.

For this purpose, we included supplementary figure 5b, which shows the extent of each of the system, mapped to the cortical surface. The text in the methods now reads:

*For each system, a larger area containing associated cortical regions was manually delineated and can be viewed mapped to the cortical surface in Supplementary Figure 5b*[*[10,17,62]*](https://paperpile.com/c/nvEgXa/UWnl6+wqehr+ezMbV)

13. Supplementary Fig. 4: The figure legend needs work. What is the meaning of the shaded sites/layers in blue/grey?

We thank the reviewer for pointing out this oversight in the figure caption. The shaded sites are manually delineated layers for comparison. The caption has now been altered to reflect this:

*Comparison of white matter surfaces generated by the neural network and by placing the white surface at the maximum intensity gradient. For visual comparison, the surfaces are overlaid on a 2D section, where manually segmented layers are available. The maximum intensity gradient white surface (red) was identified on lower-resolution data (200μm). Superimposed on the histology in grayscale is a section of cortex where six layers were manually segmented. The automated blue surface follows the manually-delineated grey-white matter boundary, which is determined by the presence of cortical neurons. By contrast the red (maximum gradient) surface follows a feature that is consistently superficial to the gray-white matter boundary, corresponding to the layer VIa/VIb boundary. This systematic difference highlights the role of using histological expertise when translating across scales and fields to ensure consistent definitions. It also raises an important question on the placement of the white surface in MRI cortical reconstructions, which is placed at the maximum MRI intensity gradient. This gradient is determined predominantly by myelin contrast, and therefore influenced by changes interregional and longitudinal in cortical myelination. Future cortical segmentation algorithms need to be developed with close reference to histological definitions of the gray/white boundary.*

Minor comments:

14. Page 3, it is not clear what the authors mean by "stereological bias".

We apologise for the lack of clarity. Stereological bias referred to the inability to measure thickness values from the entire cerebral cortex when it is sectioned in a given 2D plane. This leads to a sampling bias where only in-plane cortical areas can be sampled. We have adapted the text to clarify this.

*First, thickness measurements carried out in 2D are associated with measurement artefacts due to oblique slicing* [*[28]*](https://paperpile.com/c/nvEgXa/xnp7) *and stereological sampling bias as out-plane cortical areas cannot be included.*

15. p 10. The term "diffusive smoothing algorithm" is used, but there is no citation or explanation.

This was indeed an insufficient explanation. The text has now been expanded to explain the algorithm and a citation for the method (Kimia et al., 1996) has been included.

*The degree of anisotropic smoothing is determined by repeatedly applying the diffusive smoothing algorithm, where the degree of smoothing in a given direction is inversely related to the intensity gradient in that direction*[*[55]*](https://paperpile.com/c/nvEgXa/tD4mn)*.*

16. p 10. A diagram might help the reader understand how 'vertices' were selected and used.

We apologise for any lack of clarity in this section. A vertex is a point on the mesh surface. To generate histological intensity profiles, the methods described in this section were applied to *all* vertices on the mesh surface. We hope the following edits have helped to clarify this section:

*Pial and white surfaces originally extracted using a tissue classification of 200μm were taken as starting surfaces*[*[30]*](https://paperpile.com/c/nvEgXa/wEz6c)*. Each surface contained an equal number of surface points (“vertices”), located at corresponding anatomical locations on the two surfaces. Prior to intensity profile extraction, the vertex locations on both surfaces were altered to address several issues. First, the vectors connecting white and grey vertices were altered in order to improve their approximation of columnar trajectories and to minimise intersecting streamlines. Second, “pial” and “white” surfaces were respectively expanded beyond the pial boundary and into the white matter respectively, extending the extracted profiles to contain the whole cortex with additional padding. This was to enable the network to adjust surface placement of these borders according to features learned from the manual delineation of these boundaries.*
