# Supplementary material for: BigBrain 3D atlas of cortical layers: Cortical and laminar thickness gradients diverge in sensory and motor cortices
Source: PLoS Biol. 2020 Apr 3;18(4):e3000678. doi: 10.1371/journal.pbio.3000678 (PMC7159250; doi:10.1371/journal.pbio.3000678)
Supplement: Attachment — Submitted filename: Response_Accepted.docx [file pbio.3000678.s009.docx]

Konrad Wagstyl

Montreal Neurological Institute

McGill University

3801, rue University

Montréal, QC

Canada H3A 2B4

Tel: +44 1223 764670

Dear Editors of PLOS Biology,

We thank the reviewers and editor for their comments and accepting the manuscript.

Please find submitted the formatted manuscript entitled:

“BigBrain 3D atlas of cortical layers: cortical and laminar thickness gradients diverge in sensory and motor cortices.”

Sincerely,

Konrad Wagstyl and Alan C. Evans
